# Effect of two different primers on the shear bond strength of metallic brackets to zirconia ceramic

**DOI:** 10.1186/s12903-019-0740-6

**Published:** 2019-03-28

**Authors:** Alexander Franz, Modesto Raabe, Bledar Lilaj, Rinet Dauti, Andreas Moritz, Dieter Müßig, Barbara Cvikl

**Affiliations:** 1 Department of Conservative Dentistry and Periodontology, Medical University of Vienna, Sensengasse 2A, 1090 Vienna, Austria; 2 Department of Conservative Dentistry, Sigmund Freud University, Vienna, Austria; 3 Private practice, Gross Gerungs, Austria; 4 Centre for Orthodontics, Division of Dentistry, Danube Private University, Krems, Austria

## Abstract

**Background:**

In view of the increasing demand of adult orthodontics for esthetic purposes, adult treatment with brackets has become an important issue. One essential factor for the quality of such treatment is bracket bonding on ceramics. For testing the adhesive bond between the bracket and the ceramic surface it is important to consider the static or cyclic loading that goes along with it.

**Methods:**

Metallic Brackets were adhesively fixed on zirconia ceramic blocks in a simulated leveling phase using two different primers (Monobond S and Monobond Etch & Prime). Half of the metallic brackets were activated using a 0.14-nickel titanium wire, while the other half remained non-activated. Shear bond testing (SBT) was performed after thermocycling. Furthermore the Adhesive Remnant Index (ARI) was analyzed.

**Results:**

SBT resulted in significantly higher shear bond values when Monobond Etch & Prime was used compared to the use of Monobond S. Activation of the brackets did not show different results in comparison to the non-activated brackets. The ARI did not indicate cement remnants on the ceramic surface, regardless of the primer and the activation status.

**Conclusions:**

The use of Monobond Etch & Prime has great potential for the bonding of brackets on dental zirconia ceramics.

## Background

Adhesive systems for bonding brackets on dental hard tissue or ceramic surfaces have to meet high standards in orthodontics. The adhesive compound has to withstand the forces of orthodontic treatment and chewing movements within the moist, warm environment of the oral cavity. Furthermore, in case of a planned bracket removal, no damage should be caused on the dental hard tissue or on the dental ceramic. Studies have shown that an adhesive force between 6 and 10 Mega Pascal is required to ensure sufficient adhesion of the brackets for the acquisition and transmission of orthodontic forces without the danger of surface damage during bracket removal. [1, 2]

Thanks to intensive research and ongoing development of materials, it has become easier to achieve the required adhesive forces for bonding brackets on dental hard tissue. Successful bonding is fundamental in the most recent trends in dentistry, a discipline that has moved from a purely medical discipline focused mainly on pain relief and dental health issues to a more holistic medicine in which oral esthetics and overall appearance are of utmost importance. Within the increasing demand of bracket therapy for adult patients, the quality of bracket adhesion to ceramic restorations like crowns or veneers plays a crucial role. [3]

Since ceramics is chemically inert and hardly ever interacts with possible reactants an adhesive bond to brackets cannot be achieved with commonly used adhesive systems. [4] A chemical and/or mechanical pre-treatment of the ceramic surface is required to improve the adhesive bond between ceramic and the bracket base. This pre-treatment can be done either mechanically with a bur or a laser or by sandblasting the surface, or chemically by etching with hydrofluoric acid or phosphoric acid. [5] The best adhesion values are achieved by using hydrofluoric acid with subsequent silanization of the ceramic surface. [6–9] However, the intraoral application of hydrofluoric acid is contraindicated, since it may cause soft tissue injuries, including bone necrosis. This requires alternatives.

Furthermore, for a long-term adhesion to orthodontic brackets, hydrofluoric acid cannot adjust to all types of ceramic. Zirconia ceramics, for example, has not presented an adequately roughened and retentive surface after etching with hydrofluoric acid. [10] Within current research on the adhesive capabilities of metallic and ceramic brackets on different kinds of ceramics [7, 11, 12], no studies on zirconia ceramics can be found. This is a drawback since zirconia ceramics will play an increasingly important role in the clinical treatment of patients due to its material properties and aesthetic improvements such as a high translucency. Particularly yttrium stabilized zirconium oxide ceramics, which have been stabilized in the tetragonal phase, show strongly improved mechanical properties [13].

The tests used to assess the quality of adhesive bonds between brackets and dental hard tissue or ceramic are mainly static or cyclic shear bond tests, tensile bond tests or torsion tests. Since most in vitro systems only represent forces from one side, simulating the clinical situation with oral forces acting from different sides on the adhesive system is indeed challenging. Furthermore, within the existing testing systems for static shear bond there is no test for a possible weakness of adhesion due to chewing forces. The challenge of testing possible influences of environmental factors on cyclic shear bonding still needs to be met.

The aim of the present study was to test the shear bond strength between orthodontic brackets and dental zirconia ceramics using a new primer (Monobond Etch & Prime, Ivoclar Vivadent, Schaan, Principality of Liechtenstein) actually made for use in ceramic repairs. The results were compared with those of a conventional primer system from the same manufacturer (Monobond S, Ivoclar Vivadent). Both primers were applied without using hydrofluoric acid due to patient safety reasons. Besides the shear bond strength, possible residues of the adhesive materials either on the bracket base or on the ceramic were examined using the adhesive remnant index (ARI). A further aim of the present study was to investigate and to establish a study model that simulates intraoral forces. Artificial aging was also applied on the shear bond strength between orthodontic brackets and dental ceramics. Thus, three brackets were bonded in a simulated orthodontic leveling phase on a pretreated ceramic block. In the test group the brackets were left without any activation, while in the experimental group the brackets were activated by means of an orthodontic wire. Both groups were exposed to an artificial aging process by thermocycling. The first null hypothesis was that there is no statistically significant difference in shear bond strength between the bracket and the ceramic, regardless of the used primers. The second null hypothesis was that there is no statistically significant difference in shear bond strength between the bracket and the ceramic, regardless of whether the orthodontic wire is activated or not.

## Methods

### All materials used in this study are listed in Table 1

#### Sample preparation

**Fig. 1:**
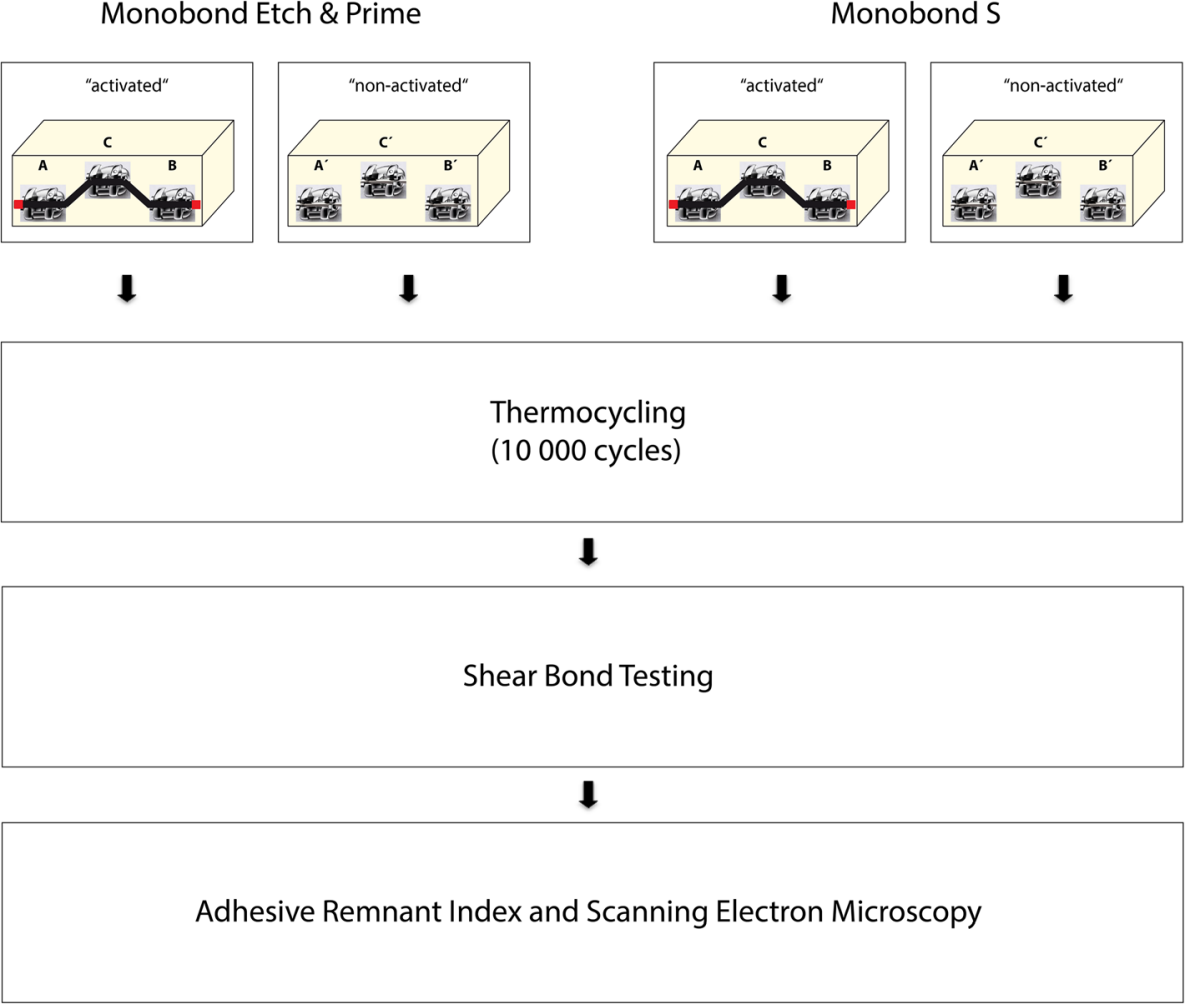
Flowchart of the experimental procedures

For the simulation of dental crowns or veneers, blocks (30 mm × 15 mm × 12 mm) of inCoris TZI zirconium oxide sinter ceramic (Dentsply Sirona, York, USA) were sintered and prepared according to the technique work steps in a dental laboratory. Subsequently, the ceramic blocks (*n* = 20) were randomized and divided into two groups and fixation of Marquis 022 Roth brackets (Ortho Technology, Tampa, USA) was done either by using the bonding agent Monobond S (Ivoclar Vivadent) or Monobond Etch & Prime (Ivoclar Vivadent). Detailed information about the bonding agents is given in Table 2. A total of 240 brackets were used in this study, 120 for the group of Monobond S and 120 for the group of Monobond Etch & Prime. In each group, half of the brackets were positioned simulating an orthodontic leveling phase using a 0.14-nickel titanium wire of specified length and rubber ligatures. To ensure that the wire remains in situ, both ends were fixed (Fig. 1, activated). The other half of the brackets was also placed simulating an orthodontic leveling phase; however, no wire was activated (Fig. 1, non-activated). Individual positions of brackets, end positions A and B, as well as the middle position C were considered in the statistical evaluation. The application of the materials (indicated in Table 1) and the bonding procedure were performed following the manufacturers recommendations. Afterwards, all specimens were subjected to thermocycling (5° Celsius and 55° Celsius, dwell time 40 s) for 10,000 cycles, simulating an intraoral period of approximately one year. [14]

**Table 1:** Materials and manufacturers

inCoris TZI zirconia ceramic	Dentsply Sirona (York, USA)
Monobond S	Ivoclar Vivadent (Schaan, Liechtenstein)
Monobond Etch & Prime	Ivoclar Vivadent (Schaan, Liechtenstein)
Transbond™ MIP	3 M ESPE (Neuss, Germany)
Light Bond™	Reliance Orthodontic Products Inc. (Illinois, USA)
Optima 10 LED	BA International Ltd. (Northampton, UK)
G4™ Nickel Titanium Archwires 0,14	OrthoForce (Levallois-Perret, France)
Rubber ligatures	SmileDental® (Ratingen, Germany)

**Table 2:** Detailed compositions of Monobond S and Monobond Etch & Prime

Monobond S:	Monobond Etch & Prime:
Silane methacrylate	Silane methacrylate
Alcohol and water	Alcohol and water
	Ammonium polyfluoride

#### Shear bond strength tests

After thermocycling, the specimens were arranged for shear tests. The orthodontic wire was removed where necessary, and the specimens were embedded in hard stone type 3 (MOLDANO® blue; Heraeus Kulzer, Hanau, Germany) by means of a plastic block. Shear bond strength tests were performed using a universal testing machine (Z010, Zwick/Roell, Ulm, Germany) with a cross head speed of 1 mm/min. The adhesion between the ceramic blocks and the brackets was recorded in mega Pascal (MPa). The debonding forces measured in Newton were used for calculating the shear bond strength in mega Pascal using the formula SBS (shear bond strength) = F (force in N) / A (cross-sectional area of the brackets in mm). For the investigation of fracture behavior, the surfaces of the sheared off brackets as well as the ceramic blocks were evaluated using reflected light microscopy. On a random basis, three samples of each group were examined using SEM. The Adhesive Remnant Index (ARI) recording to Artun and Bergland was used for tracking cement residues on the ceramic surfaces. [4, 15] An ARI score of 0 represents no cement residues, a score of 1 means less than 50% of cement residues, a score of 2 means more than 50% cement residues, and a score of 3 represents that the entire cement remains on the ceramic surface.

#### Statistical methods

Statistical analysis was performed using SPSS 19.0. The data of the shear bond test were analyzed using the non-parametric Mann-Whitney-U Test due to the absence of a normal distribution. The level of significance was set at *p* < 0,05. The data concerning the adhesive remnant index were descriptively presented.

## Results

### Adhesion of brackets using two different bonding agents with or without the activation of the brackets

**Fig. 2:**
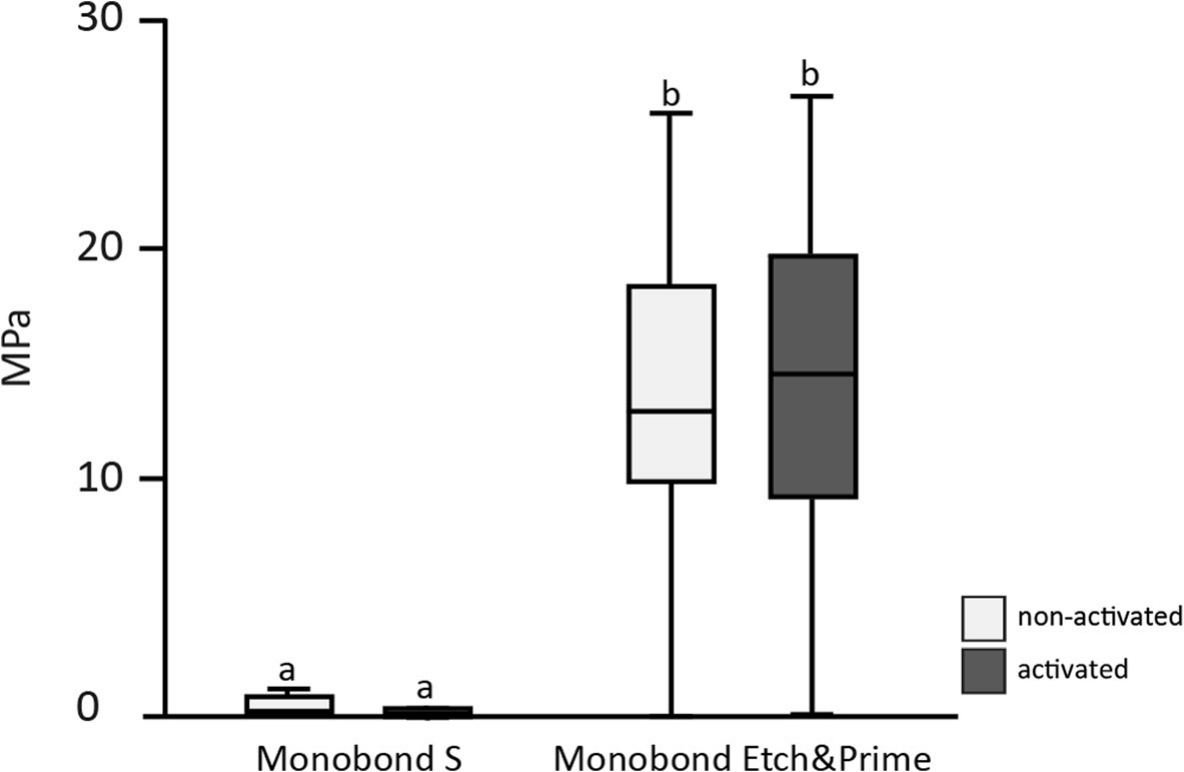
Shear bond strength between brackets and dental ceramic using Monobond S or Monobond Etch & Prime with/without activation of brackets using a 0.14-nickel titanium wire

The adhesive fixation of common metallic brackets on zirconium oxide using Monobond Etch & Prime as primer resulted in shear bond strength values statistically more significant than those resulting from the use of Monobond S as primer (p < 0,05) (Fig. 2). This effect was observed for brackets activated using a 0.14-nickel titanium wire as well as for brackets without activation. Results of shear bond tests in mean, standard deviation and coefficient of variation are given in Table 3.

**Table 3:** Results of shear bond tests in mean, standard deviation and coefficient of variation

Monobond S non-activated	Monobond S activated	Monobond Etch&Prime non-activated	Monobond Etch&Prime activated
mean: 0.73	mean: 0.33	mean: 13.66	mean: 14.53
standard deviation: 1.71	standard deviation: 1.31	standard deviation: 5.97	standard deviation: 6.22
coefficient of variation:2.3	coefficient of variation: 3.9	coefficient of variation: 0.4	coefficient of variation: 0.4

Regarding the activation with the 0.14-nickel titanium wire, shear bond strength values for Monobond S were significantly higher after the activation of the brackets than without activation (*p* = 0,002). Also in the group using Monobond Etch & Prime higher shear bond strength values for activated brackets could be determined, although differences from brackets without activation were not statistically significant (*p* > 0,05).

A higher amount of pretest failures was observed for the group using Monobond S as a primer compared to the group using Monobond Etch & Prime. For Monobond Etch & Prime 119 out of 120 specimens could be tested, regardless of whether specimens were activated with a wire or not. For brackets bonded with Monobond S and activated with a 0.14-nickel titanium wire, only four out of 60 specimens could be tested; for brackets bonded with Monobond S without activation a total of 18 (out of 60) specimens could be tested.

### Influence of individual positions and activation of brackets on shear bond strength values

The individual position of brackets (end position or middle position) had no statistically significant influence on the bracket shear bond strength. This was the case for Monobond S and Monobond Etch & Prime, regardless of activation of brackets (data not shown).

### Adhesive remnant index (ARI) using reflective light microscopy

No fractures in the ceramic blocks were observed in any of the primer materials. In the Monobond S group only one specimen showed an ARI score greater than 0 on the ceramic surface. In the Monobond Etch & Prime group cement residues on the ceramic surface were only sporadically observed, however never exceeding an ARI score of 1 (Table 4).

**Table 4:** Adhesive Remnant Index indicating cement residues on the ceramic surface; ARI 0: no cement residues on the ceramic surface, ARI 1: less than 50% of cement residues on the ceramic surface, ARI 2: more than 50% of cement residues on the ceramic surface, ARI 3: ceramic surface completely covered with cement

	ARI 0	ARI 1	ARI 2	ARI 3
Monobond S	119	1	0	0
Monobond Etch & Prime	114	6	0	0

### Investigation of samples using SEM

**Fig. 3:**
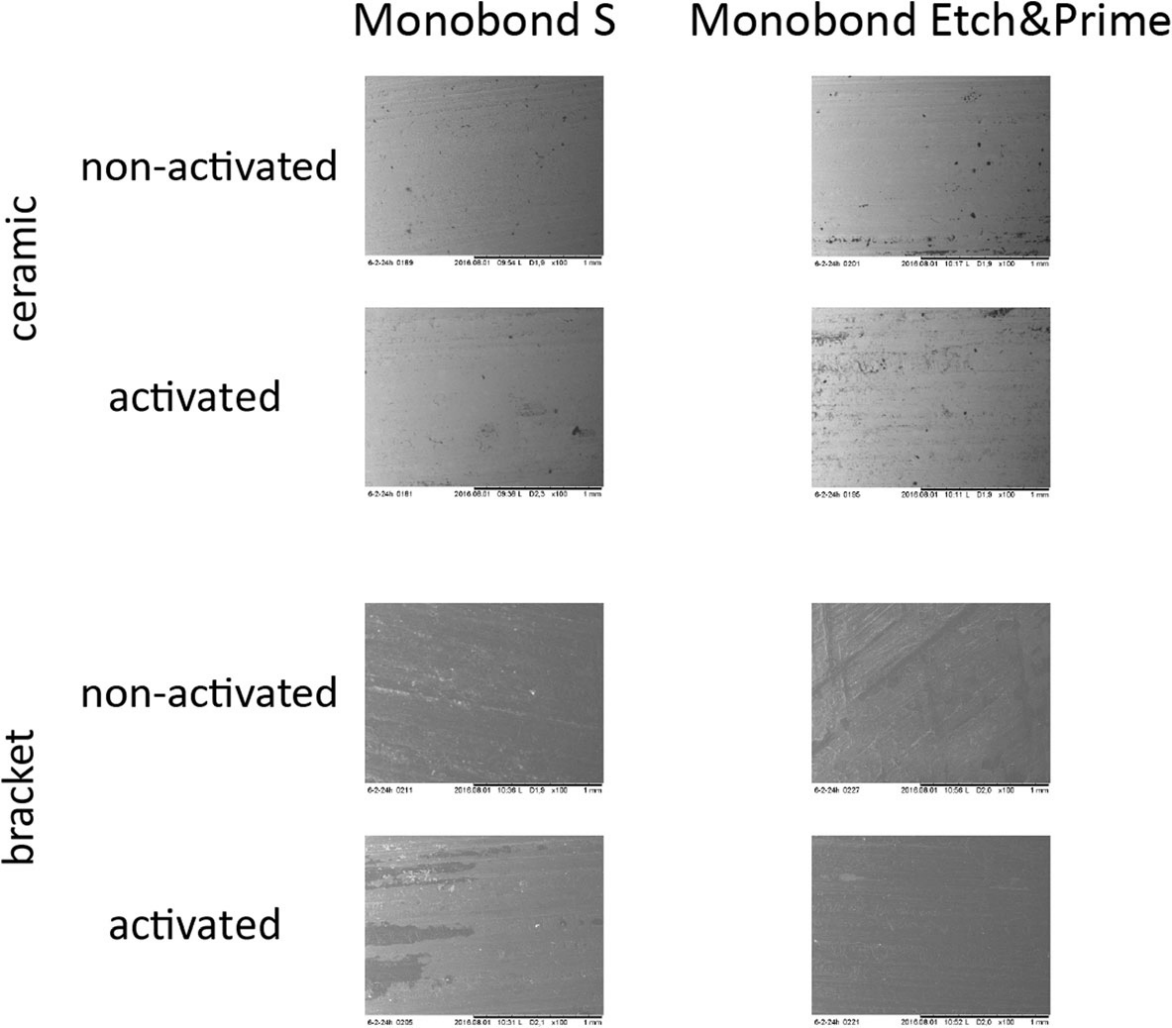
Scanning electron microscopy images of three randomized samples for each group. No signs of fracture could be detected on the ceramic surfaces, regardless of both the primer and the activation. Residues of the cement are only visible on the bracket basis, regardless of both the primer and the activation

On a random basis three ceramic surfaces and the corresponding bracket basis of each group (bonding agent, wire-activation and position of the bracket) were investigated using SEM (Fig. 3). Images of scanning electron microscopy confirmed the results of Adhesive Remnant Index classification by reflective light microscopy.

## Discussion

Due to the increasing need for not only healthy but also esthetically pleasing teeth, the demand of adult orthodontics has massively increased in recent years. This positive trend also poses a problem for orthodontists, since orthodontic retention elements such as brackets not only have to be fixed on dental hard tissue, but also on dentures such as dental ceramics. However, since dental ceramics, by virtue of their nature, do not bind with commercial adhesive systems per se, ways have to be found to ensure adequate adhesion of the brackets. The aim of the present study was on the one hand to test two different primers for adhesive fixation in orthodontic treatment, and on the other hand to test an in vitro model simulating the clinical intraoral situation with simultaneous exposure to heat, cold and the forces transmitted through a single wire.

In summary, it can be said that the adhesive forces achieved between the dental ceramic and the bracket when using the primer Monobond Etch & Prime were significantly higher than when using the primer Monobond S. Most brackets in the Monobond S group already fell off in the course of the artificial aging process, whereas only 2 of the 120 brackets in the Monobond Etch & Prime group fell off during artificial aging. This high failure rate in the Monobond S group further explains the large standard deviation and consequently the high coefficient of variation. Thus, the first null hypothesis, that is, that no statistically significant difference in shear bond strength between the bracket and the ceramic occurs regardless of the used primers, Monobond Etch & Prime or Monobond S, can be rejected.

The second null hypothesis, that is, that there is no statistically significant difference in shear bond strength between the wire-activated test group and the non-activated control group has to be rejected for the Monobond S group but has to be accepted for the Monobond Etch & Prime group. However, it should be noted that due to the low adhesion values of less than 1 MPa found in this study when using Monobond S without hydrofluoric acid, this statistical finding is irrelevant. Hence, a clinical use with these low adhesion values cannot be recommended. With regard to the different positions of the brackets in the simulated leveling phase of the orthodontic treatment, end position versus position in the middle, the null hypothesis has to be accepted, regardless of the adhesive material used. Thus, there are no different shear bond values when the bracket is subjected to a force from two sides (the bracket in the middle), or only from one side (the bracket on the end). This finding might seem faulty from the physical point of view, but it might be due to the low forces exerted by a 0.14-nickel titanium wire simulating the leveling phase.

The present study concurs with the state of the art in the use of Monobond Etch & Prime for adhesion of brackets on dental ceramic, showing that shear bond values of 13 to 14 MPa were achieved without damaging the ceramic surface when debonding was performed, which is absolutely necessary in daily clinical practice. [15] The fact that these adhesion values were achieved without pretreatment with hydrofluoric acid and after a simulated intraoral period of one year is of particular clinical interest. Due to its possible hazardous effects, hydrofluoric acid is contraindicated, since it is toxic and can rapidly penetrate into deep tissue layers causing massive tissue damage, including bone necrosis. Therefore, it can only be applied on extra-oral areas. [16]

It is also worth mentioning that in our study no fractures in the ceramic occurred when the brackets were actively debonded using the universal testing machine. The use of Monobond Etch & Prime resulted in shear bond strength values between the brackets and the zirconia ceramic that lay within the perfect range for orthodontic treatment. Less than 6 MPa results in bracket loss during treatment and more than 13 MPa might result in cohesive fracture of the ceramic during debonding. [17] However, only shear bond values higher than 13 MPa might result in ceramic fracture when zirconia ceramics are used.

No shear bond values reaching the clinically required 6–10 MPa were achieved in the use of Monobond S for bracket adhesion on the dental ceramic. Furthermore, most brackets had already loosened during the artificial aging process, so that they did not reach a year’s lifespan during the simulated intraoral period of one year. Whether pretreatment of the dental ceramic with hydrofluoric acid would have led to better shear bond values is of course an important issue to be investigated in future studies. This is of particular interest since the dental ceramic used was not a glass ceramic, which would react adequately to an etching with hydrofluoric acid. [3, 4] The clinical relevance of such a study must, of course, be questioned, since as already explained, hydrofluoric acid should not be used in the oral cavity.

In the present study yttrium stabilized zirconium oxide ceramics were used to simulate the clinical situation. The uniqueness of these ceramics lies in their outstanding mechanical properties paired with a meanwhile very high aesthetic quality. With these stabilized zirconium oxide ceramics the so-called tetragonal phase can be stabilized and there is no transformation into the monoclinic phase, which has poorer mechanical properties, but would be the more natural phase at room temperature. However, these outstanding mechanical properties make the classical pretreatment of ceramics for the adhesive bond aimless. It is therefore so intriguing that two primers, of which the presence or absence of ammonium polyfluoride is the only difference, lead to such different results in shear bond strength. Ammonium polyfluoride might lead to a change of the ceramic surface, which explains the better bond. Whether there is a transformation of the different phases, from tetragonal to monoclinic for example, cannot be answered on the basis of the present study. The analysis of the structure of yttrium stabilized zirconium oxide ceramics after application of ammonium polyfluoride would be however of great interest for future studies. However, the clinical performance of Monobond Etch & Prime has been confirmed in numerous studies, but with the difference that other ceramics such as glass ceramics were used [18–20]. Furthermore, to our knowledge, Monobond Etch & Prime was never used for bonding brackets on yttrium stabilized zirconium oxide ceramics.

The implementation of the amount of 10,000 cycles is questionable, since most brackets bonded with Monobond S could not be tested due to pretesting-failures during thermocycling. In previous studies on bond strengths between brackets and ceramic surfaces no thermocycling at all [21, 22] or only up to 500 cycles [6, 7] were applied. However, there is evidence that applying a higher number of cycles reflects more closely the clinical situation, in which brackets need to be adhered throughout the whole orthodontic treatment. It is well known that mechanical properties decrease due to aging [13], nevertheless the influence of a simulated orthodontic treatment was not that influencing as expected. This might be due to the relative low forces at the simulated leveling phase. When using Monobond Etch & Prime, 118 of 120 brackets remained on the ceramic surface after applying 10,000 cycles and achieved shear bond strength values suitable for clinical practice. These values are comparable to the results of a study in which also 10,000 cycles were applied, but with previous etching using hydrofluoric acid. [16]

Another aim of our study was to find out if the integration of an activated orthodontic wire might influence the bond between brackets and ceramic surfaces, or if the force caused by the wire is too low to negatively affect the shear bond strength. This experimental approach is novel, since the testing of adhesion is hitherto carried out using mainly static tests or cyclic shear-, tensile- or torsion tests. [23, 24] The adhesion of brackets is burdened from several sides, alongside with changing temperature conditions in a moist, warm environment within the intra-oral environment. Nevertheless, no significant results between brackets activated with wire compared to brackets without activation could be found.

The major limitation of our study is the difficulty to compare an in vitro study with the conditions in clinical practice. Furthermore, we used materials that were not directly developed for our research question. Nevertheless, this could be a strong point of the present study, since it reveals possible new characteristics of these materials. Another limitation of our study could be the fact that almost none of the brackets bonded with Monobond S could be tested due to initial failures, so that no evidence was obtained on the possible bonding durability without artificial aging.

## Conclusions

1. High shear bond strength values above the optimal range of 6–8 MPa for orthodontic treatment were detected between metal brackets and zirconium oxide ceramic when applying Monobond Etch & Prime for fixation.
2. Low shear bond strength values missing the optimal range of 6–8 MPa for orthodontic treatment were detected between metal brackets and zirconium oxide ceramic when applying Monobond S for fixation.
3. Activation of the brackets with a 0.14-nickel titanium wire did not result in clinically relevant differences.
4. Different positions of the brackets in the simulated leveling phase had no statistically significant influence on shear bond strength values.
5. No cohesive fracture in the ceramic occurred, regardless of the used primer.
